# Equity and Longitudinal Assessments: Perspectives from Physician Assistants/Associates (PAs) Participating in PANRE-LA

**DOI:** 10.1007/s40670-025-02329-4

**Published:** 2025-02-27

**Authors:** Andrzej Kozikowski, Joshua Goodman, Andrew Dallas, Yanlin Jiang

**Affiliations:** National Commission on Certification of Physician Assistants, 12000 Findley Road, Suite 200, Johns Creek, GA 30097 USA

**Keywords:** Longitudinal assessments, Maintenance of certification, Equity, Physician assistants/associates

## Abstract

**Introduction:**

Longitudinal assessments (LAs) may offer more flexibility and unique opportunities to enhance equity. Although prior findings on LAs demonstrate that they foster learning, limited research exists on potential differences in examinee perspectives by demographics and practice characteristics. Addressing this research gap is vital to ensuring that examinees from different backgrounds equally derive learning benefits from LAs.

**Methods:**

We evaluated potential differences in perspectives and experiences of physician assistants/associates (PAs) participating in the PA National Recertifying Examination Longitudinal Assessment (PANRE-LA) program, considering a wide range of demographic and practice characteristics.

**Results:**

Over 90% agreed that this type of assessment provides a learning experience, helps to identify gaps, provides an opportunity to improve, aligns with a lifelong learning perspective, and keeps core medical knowledge up-to-date. Approximately 84% believed it helps them to be a better practitioner, and 78.7% either anticipated or had applied learning from PANRE-LA to their clinical practice.

**Discussion:**

Our findings suggest that PAs across diverse demographics and practice characteristics equally derive self-reported learning benefits from PANRE-LA. LAs, due to their formative components, may provide unique opportunities to promote equity in knowledge acquisition, foster continuous learning, and ultimately contribute to improved patient care.

Historically, certifying boards relied on secure summative point-in-time standardized assessments for maintenance of certification (MOC). These high-stakes assessments undergo a rigorous development process to ensure they provide reliable and valid inferences regarding whether examinees maintain the minimum competence level required for patient care. Differential item functioning (DIF) studies can evaluate them for possible bias, and an independent and diverse panel of experts can review test content [1, 2]. However, examinees have regarded single-point-in-time assessments unfavorably, considering them anxiety-inducing and suboptimal for assessing competence or promoting learning [3, 4]. Moreover, research shows concerning associations between examinee characteristics and performance on summative point-in-time MOC assessments [5–10]. Factors such as increasing years in practice or older age have been consistently linked with lower performance. Providers further from their initial training or those who have specialized may struggle to recall information less frequently used in their current practice.

Additionally, the rate of change in standards of care continues to accelerate [11], requiring providers to not only rapidly learn and adopt new standards but also discard outdated practices [12]. This dynamic environment further underscores traditional MOC assessments’ limitations in catering to providers’ diverse learning needs. Furthermore, the high-stakes nature of these exams can exacerbate test anxiety, potentially leading to poorer performance for some groups of examinees [13, 14].

LAs are based on adult learning theory and may offer more flexibility and unique opportunities to enhance equity. Similar to summative single-point-in-time assessments, LAs can be evaluated for bias by DIF studies, and an independent panel of experts can review test content. However, LAs go beyond single-point-in-time MOC assessments because they incorporate formative (assessment for learning) components. The formative aspects of LAs are based on advances in cognitive psychology, including spaced learning, more frequent testing, immediate feedback, and interleaving [13]. These aspects are designed to provide a learning experience, help providers identify knowledge gaps, provide opportunities to improve, help to keep up to date, and foster life-long learning. These core components could potentially help mitigate existing inequities.

Certification assessments began shifting to LAs in 2014 when the American Board of Anesthesiology started piloting its Maintenance of Certification in Anesthesiology program [4]. Other medical boards followed suit [15–17], and now all American Board of Medical Specialties (ABMS) member boards offer LAs for MOC [18]. Similarly, after completing a 2-year pilot, the National Commission on Certification of Physician Assistants (NCCPA) launched in 2023 the Physician Assistant National Recertifying Examination Longitudinal Assessment (PANRE-LA). The new recertification pathway was presented as a permanent alternative to the traditional PANRE, a high-stakes single-point-in-time 5-h summative exam completed in a secure testing center every 10 years. Although LAs are conceptualized and designed in diverse ways by different certifying organizations, they have common elements that promote learning, including being spaced over time, identifying knowledge gaps by providing examinees with feedback with rationales for correct and incorrect responses, retesting knowledge gaps, and providing resources for further learning [19]. LAs are administered online, can be completed at home, and allow for the use of resources to help answer questions in a limited time period per question [19].

Equity in assessment can be approached from various perspectives, with a fairness orientation being one of the most commonly adopted [14, 20]. A fairness-oriented view of equity in assessment involves providing equal access to learning opportunities and ensuring that assessments are unbiased [14, 21]. Both the traditional PANRE and PANRE-LA undergo rigorous psychometric development processes to ensure fairness. However, PANRE-LA offers unique features that may enhance equity by increasing access to learning opportunities. Traditional exams provide limited feedback, leaving test takers uncertain about areas for improvement. In contrast, PANRE-LA offers immediate, actionable feedback, empowering participants to learn throughout the process and promoting equity in professional development. These attributes may be particularly beneficial for those further from their initial training or those who have specialized and struggle to recall information less frequently used in their current practice. Traditional exams are high-stakes and time-limited, which may disadvantage individuals who experience test anxiety or struggle in high-pressure settings, which can affect their performance. Conversely, PANRE-LA divides knowledge assessments into smaller, less stressful increments over time. Additionally, traditional exams typically require significant preparation, often involving expensive prep courses or materials, which may be out of reach for individuals with limited resources. PANRE-LA integrates learning into the assessment process through its formative components, potentially reducing reliance on external preparation. Traditional assessments are taken in testing centers, requiring time off from work and expenses related to travel. This can be challenging for individuals living in rural areas and those with family responsibilities and limited resources. PANRE-LA may be more accessible as exam items can be completed in the comfort of their homes.

Research investigating perspectives regarding different LA programs has been conducted with certified registered nurse anesthetists (CRNAs) [22], pediatricians [23], family physicians [24, 25], and anesthesiologists [4]. A 2023 scoping review by Ward et al. suggests that using LAs for certification maintenance is in the early stages [19]. The authors note that diplomates’ overall perceptions of LAs are favorable in that the process helps keep knowledge up to date and fosters life-long learning. Most ABMS certifying boards gather demographic data and practice information from their diplomates [26]. Similarly, the NCCPA collects this information from physician assistants/associates (PAs) [27, 28]. These data collection efforts allow certifying boards to identify and address potential biases in certification processes, ensure fair representation, and tailor programs to meet the diverse needs of providers and their patients.

However, limited research exists on potential differences in examinee perspectives regarding LAs based on demographics and practice characteristics. This research is vital to determine whether examinees from different backgrounds equally derive learning benefits from LAs. Given this research gap, we evaluated the perspectives and experiences of PAs participating in PANRE-LA and assessed whether views vary based on a broad range of demographic and practice characteristics.

## Methods

**Fig. 1:**
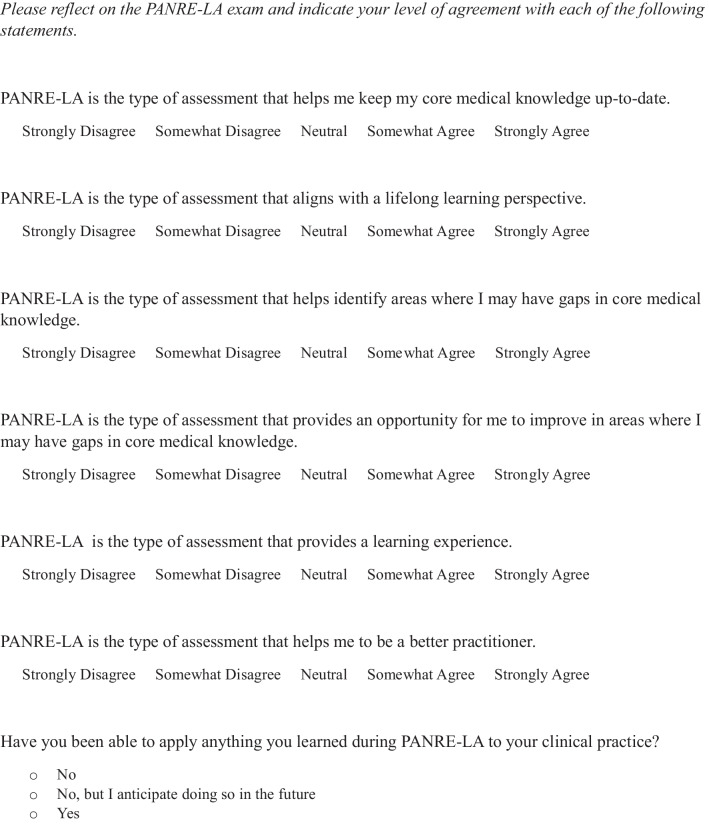
Survey items regarding perspectives and applying learning from PANRE-LA to clinical practice

This cross-sectional study was determined to be exempt by Sterling IRB (#11,939) and utilized data gathered from a larger program evaluation online survey of participants in the PANRE-LA. This survey focused on PAs’ initial experiences and perspectives and was launched after they completed the first two quarters of the PANRE-LA program. We developed the items to elicit PA views regarding PANRE-LA fostering learning and whether they have been able to use what they learned during the program in their clinical practice. We followed a robust iterative questionnaire development process, which included adapting and refining previously used items and developing new ones based on a comprehensive literature review. Items were evaluated for consistency with the study purpose, clarity, conciseness, and relevance. The final set of items used in the present analyses is presented in Fig. 1.

To decrease the number of questions on the survey, we obtained demographic and practice characteristics from the PA Professional Profile [27, 28] and merged them with survey responses using a unique identifier in both datasets. These variables were used to investigate potential differences in perspectives on PANRE-LA, including age, gender, race, ethnicity, speaking a second language, US region, rural–urban setting, specialty changes, and practice area.

The survey was programmed in Qualtrics and extensively tested for question flow, navigation, and completion time. The finalized version was launched on June 27, 2023, with data collection continuing until July 18, 2023. Three reminders with 1-week intervals were sent to PAs who still needed to complete the survey. There were 45,123 PAs who were active in Q1 and Q2 of 2023; 2226 PAs in this group had opted not to receive surveys from NCCPA. Thus, the survey was sent to 42,897 PAs; 11,896 participated for a 27.7% response rate. Further, 10,920 (91.8%) completed the survey without skipping any questions.

We conducted descriptive statistics (mean [SD], counts, percentages, as appropriate) of all variables. To simplify interpretation, we collapsed the five-category agreement scale evaluating PANRE-LA perspectives into two mutually exclusive categories: “agree” (combining “strongly agree” and “agree”) and “disagree/neutral” (encompassing all other responses). We conducted bivariate analyses (chi-square, *t*-tests, ANOVAs with follow-up Scheffe tests, as appropriate) to determine if any PA characteristics were significantly associated with perspectives regarding PANRE-LA and whether PAs have applied learning from the program to their clinical practice.

Additionally, the applying to learning question response options were dichotomized into “no” and “no, but anticipate doing so in the future/yes” so that a multivariate logistic regression could be performed. To ensure that multicollinearity was not an issue (e.g., age and years in practice) in the multivariate logistic regression, we computed the variance inflation factor (VIF). All VIF values were well under 5, indicating the absence of multicollinearity [29]. All analyses were conducted using R version 4.2.3 (R Foundation for Statistical Computing, Austria). Statistical significance was set at *p* < 0.05; all tests were two-tailed.

## Results

Table 1 provides the demographic and practice characteristics of the sample. The mean age and years certified (a proxy for the number of years in practice) were 53.0 and 23.7, respectively. The majority were female (67.3%), white (86.6%), and residing in an urban setting (91.6%). Approximately 6% self-identified as Hispanic/Latino(a), and 22.0% said they speak a second language in addition to English with their patients. The highest proportion reported to reside in the South (34.7%), followed by the Northeast (24.8%), West (20.9%), and Midwest (19.6%). A substantial proportion (68.8%) changed their specialty at least once during their career, and the top three most prevalent disciplines were primary care (24.2%), surgery-subspecialties (19.6%), and internal medicine-subspecialties (10.4%).

**Table 1:** PA characteristics, perspectives on PANRE-LA and application of learning to clinical practice

Characteristic		All (*N* = 11,896)
Age	Mean (SD)	53.0 (7.3)
Gender	Female	7997 (67.3%)
	Male	3888 (32.7%)
Race	White	9739 (86.6%)
	Asian	506 (4.5%)
	Black/African American	422 (3.8%)
	Other	584 (5.2%)
Ethnicity	Non-Hispanic/Latino(a)	10,620 (94.2%)
	Hispanic/Latino(a)	650 (5.8%)
Speaks second language	No	8992 (78.0%)
	Yes	2529 (22.0%)
US region	South	4122 (34.7%)
	Northeast	2945 (24.8%)
	West	2477 (20.9%)
	Midwest	2321 (19.6%)
Rural–urban setting	Urban	10,838 (91.6%)
	Rural/isolated	994 (8.4%)
Years certified	Mean (SD)	23.7 (5.5)
Changed specialties	Did not change	2614 (31.2%)
	Changed one or more times	5776 (68.8%)
Specialty	Primary care	2594 (24.2%)
	Surgery-subspecialties	2103 (19.6%)
	Internal medicine — subspecialties	1111 (10.4%)
	Emergency medicine	962 (9.0%)
	Dermatology	593 (5.5%)
	Surgery-general	290 (2.7%)
	Hospital medicine	208 (1.9%)
	Psychiatry	179 (1.7%)
	Critical care medicine	118 (1.1%)
	Other	2576 (24.0%)
Provides a learning experience	Disagree/neutral	771 (6.9%)
	Agree	10,372 (93.1%)
Helps identify areas where I may have gaps in core medical knowledge	Disagree/neutral	857 (7.7%)
	Agree	10,296 (92.3%)
Provides an opportunity to improve in areas where I may have gaps in core medical knowledge	Disagree/neutral	928 (8.3%)
	Agree	10,216 (91.7%)
Aligns with a lifelong learning perspective	Disagree/neutral	994 (8.9%)
	Agree	10,158 (91.1%)
Helps me keep my core medical knowledge up-to-date	Disagree/neutral	1089 (9.8%)
	Agree	10,067 (90.2%)
Helps me to be a better practitioner	Disagree/neutral	1763 (15.8%)
	Agree	9373 (84.2%)
Have you been able to apply anything you learned during PANRE-LA to your clinical practice?	No	2389 (21.3%)
	No, but I anticipate doing so in the future	4572 (40.7%)
	Yes	4273 (38.0%)

As depicted in Table 1, the highest proportion (93.1%) agreed that this type of assessment provides a learning experience, followed by that it helps identify gaps in core medical knowledge (92.3%), and that it provides an opportunity to improve in areas that PAs may have gaps in core medical knowledge (91.7%), aligns with a lifelong learning perspective (91.1%), and keeps core medical knowledge up-to-date (90.2%). However, slightly fewer (84.2%) believed it helps them to be a better practitioner. When asked whether PAs have been able to apply anything they learned during PANRE-LA to their clinical practice, the responses were more mixed: 21.3% had not, 40.7% anticipated doing so in the future, and 38.0% confirmed they had already done so.

Table 2 provides detailed results of bivariate analyses exploring potential differences by the ten PA demographic and practice characteristics on agreement whether PANRE-LA is a type of assessment that provides a learning experience, helps identify gaps in core medical knowledge, and provides an opportunity to improve in areas where PAs may have gaps in core medical knowledge. None of the associations were statistically significant (all *p* > 0.05), suggesting consensus in agreement irrespective of demographic group or practice area. Similarly, as seen in Table 3, there were no significant associations between PA demographics and practice characteristics on the likelihood of agreeing that PANRE-LA is a type of assessment that aligns with life-long learning, helps to keep core medical knowledge up-to-date, and helps to be a better practitioner.

**Table 2:** PA demographic and practice characteristics by perspectives regarding PANRE-LA

Characteristic		Provides a learning experience			Helps identify areas where I may have gaps in core medical knowledge			Provides an opportunity for me to improve in areas where I may have gaps in core medical knowledge		
		Disagree/neutral (*n* = 771)	Agree (*n* = 10,372)	*p*-value	Disagree/neutral (*n* = 857)	Agree (*n* = 10,296)	*p*-value	Disagree/neutral (*n* = 928)	Agree (*n* = 10,216)	*p*-value
Age	Mean (SD)	53.2 (7.3)	53.2 (7.4)	0.857	53.1 (7.1)	53.2 (7.4)	0.742	53.2 (7.0)	53.2 (7.4)	0.866
Gender	Female	544 (7.2%)	6968 (92.8%)	0.063	594 (7.9%)	6924 (92.1%)	0.244	637 (8.5%)	6876 (91.5%)	0.448
	Male	227 (6.3%)	3395 (93.7%)		263 (7.3%)	3363 (92.7%)		291 (8.0%)	3331 (92.0%)	
Race	White	628 (6.9%)	8486 (93.1%)	0.677	695 (7.6%)	8427 (92.4%)	0.558	750 (8.2%)	8363 (91.8%)	0.547
	Asian	31 (6.5%)	445 (93.5%)		38 (8.0%)	438 (92.0%)		46 (9.7%)	430 (90.3%)	
	Black/African American	31 (7.7%)	370 (92.3%)		32 (8.0%)	369 (92.0%)		34 (8.5%)	367 (91.5%)	
	Other	44 (8.0%)	505 (92.0%)		51 (9.3%)	499 (90.7%)		52 (9.5%)	498 (90.5%)	
Ethnicity	Non-Hispanic/Latino(a)	687 (6.9%)	9260 (93.1%)	0.950	767 (7.7%)	9187 (92.3%)	0.944	828 (8.3%)	9119 (91.7%)	0.912
	Hispanic/Latino(a)	43 (7.1%)	566 (92.9%)		46 (7.5%)	564 (92.5%)		52 (8.5%)	557 (91.5%)	
Speaks Second Language	No	592 (7.0%)	7813 (93.0%)	0.335	656 (7.8%)	7756 (92.2%)	0.649	697 (8.3%)	7707 (91.7%)	1.000
	Yes	154 (6.5%)	2234 (93.6%)		179 (7.5%)	2211 (92.5%)		198 (8.3%)	2191 (91.7%)	
US Region	South	265 (6.8%)	3608 (93.2%)	0.571	290 (7.5%)	3586 (92.5%)	0.056	301 (7.8%)	3571 (92.2%)	0.072
	Northeast	178 (6.5%)	2583 (93.6%)		189 (6.8%)	2577 (93.2%)		215 (7.8%)	2547 (92.2%)	
	West	169 (7.4%)	2127 (92.6%)		182 (7.9%)	2115 (92.1%)		204 (8.9%)	2093 (91.1%)	
	Midwest	158 (7.2%)	2028 (92.8%)		194 (8.9%)	1993 (91.1%)		206 (9.4%)	1980 (90.6%)	
Rural–urban setting	Urban	714 (7.0%)	9424 (93.0%)	0.170	780 (7.7%)	9367 (92.3%)	0.861	838 (8.3%)	9301 (91.7%)	0.363
	Rural/isolated	55 (5.8%)	893 (94.2%)		75 (7.9%)	874 (92.1%)		87 (9.2%)	861 (90.8%)	
Years certified	Mean (SD)	24.2 (5.7)	24.0 (5.5)	0.274	24.1 (5.6)	24.0 (5.5)	0.644	23.9 (5.5)	24.0 (5.6)	0.823
Changed specialties	Did not change	150 (6.2%)	2262 (93.8%)	0.207	181 (7.5%)	2232 (92.5%)	0.993	189 (7.8%)	2221 (92.2%)	0.508
	Changed	381 (7.0%)	5042 (93.0%)		409 (7.5%)	5018 (92.5%)		451 (8.3%)	4972 (91.7%)	
Practice area	Primary care	179 (7.3%)	2266 (92.7%)	0.140	212 (8.7%)	2236 (91.3%)	0.395	212 (8.7%)	2231 (91.3%)	0.617
	Surgery — subspecialties	129 (6.6%)	1815 (93.4%)		151 (7.8%)	1794 (92.2%)		163 (8.4%)	1782 (91.6%)	
	Emergency medicine	46 (5.1%)	860 (94.9%)		69 (7.6%)	837 (92.4%)		63 (7.0%)	842 (93.0%)	
	Internal medicine — subspecialties	77 (7.4%)	962 (92.6%)		73 (7.0%)	967 (93.0%)		86 (8.3%)	954 (91.7%)	
	Dermatology	35 (6.4%)	511 (93.6%)		45 (8.2%)	501 (91.8%)		46 (8.4%)	501 (91.6%)	
	Surgery — general	13 (4.8%)	258 (95.2%)		14 (5.2%)	257 (94.8%)		16 (5.9%)	255 (94.1%)	
	Hospital medicine	15 (7.9%)	175 (92.1%)		10 (5.3%)	180 (94.7%)		16 (8.4%)	174 (91.6%)	
	Psychiatry	18 (10.7%)	150 (89.3%)		11 (6.5%)	159 (93.5%)		19 (11.2%)	151 (88.8%)	
	Critical care medicine	5 (4.6%)	104 (95.4%)		6 (5.5%)	103 (94.5%)		7 (6.5%)	101 (93.5%)	
	Other	175 (7.3%)	2238 (92.7%)		180 (7.5%)	2236 (92.5%)		207 (8.6%)	2208 (91.4%)

**Table 3:** PA demographic and practice characteristics by perspectives regarding PANRE-LA

Characteristic		Aligns with a lifelong learning perspective			Helps me keep my core medical knowledge up-to-date			Helps me to be a better practitioner		
		Disagree/neutral (*n* = 994)	Agree (*n* = 10,158)	*p*-value	Disagree/neutral (*n* = 1089)	Agree (*n* = 10,067)	*p*-value	Disagree/neutral (*n* = 1763)	Agree (*n* = 9373)	*p*-value
Age	Mean (SD)	53.2 (7.2)	53.2 (7.4)	0.946	53.2 (7.2)	53.2 (7.4)	0.853	53.4 (7.2)	53.2 (7.4)	0.237
Gender	Female	696 (9.3%)	6820 (90.7%)	0.076	746 (9.9%)	6771 (90.1%)	0.449	1207 (16.1%)	6298 (83.9%)	0.336
	Male	298 (8.2%)	3329 (91.8%)		343 (9.5%)	3287 (90.6%)		556 (15.4%)	3066 (84.6%)	
Race	White	816 (8.9%)	8308 (91.1%)	0.718	885 (9.7%)	8241 (90.3%)	0.245	1446 (15.9%)	7661 (84.1%)	0.742
	Asian	37 (7.8%)	438 (92.2%)		39 (8.2%)	437 (91.8%)		75 (15.8%)	400 (84.2%)	
	Black/African American	40 (10.0%)	360 (90.0%)		46 (11.5%)	355 (88.5%)		72 (18.0%)	329 (82.0%)	
	Other	48 (8.7%)	501 (91.3%)		62 (11.3%)	487 (88.7%)		88 (16.0%)	462 (84.0%)	
Ethnicity	Non-Hispanic/Latino	889 (8.9%)	9066 (91.1%)	0.881	976 (9.8%)	8985 (90.2%)	0.796	1588 (16.0%)	8351 (84.0%)	0.920
	Hispanic/Latino	56 (9.2%)	553 (90.8%)		57 (9.4%)	550 (90.6%)		96 (15.7%)	514 (84.3%)	
Speaks second language	No	756 (9.0%)	7658 (91.0%)	0.665	839 (10.0%)	7577 (90.0%)	0.192	1354 (16.1%)	7044 (83.9%)	0.121
	Yes	207 (8.7%)	2180 (91.3%)		216 (9.1%)	2172 (91.0%)		353 (14.8%)	2035 (85.2%)	
US region	South	350 (9.0%)	3525 (91.0%)	0.570	363 (9.4%)	3516 (90.6%)	0.230	591 (15.3%)	3281 (84.7%)	0.104
	Northeast	230 (8.3%)	2537 (91.7%)		263 (9.5%)	2505 (90.5%)		415 (15.0%)	2343 (85.0%)	
	West	207 (9.0%)	2089 (91.0%)		223 (9.7%)	2072 (90.3%)		377 (16.4%)	1918 (83.6%)	
	Midwest	206 (9.4%)	1981 (90.6%)		239 (10.9%)	1948 (89.1%)		377 (17.3%)	1807 (82.7%)	
Rural–urban setting	Urban	913 (9.0%)	9233 (91.0%)	0.456	995 (9.8%)	9155 (90.2%)	0.960	1602 (15.8%)	8529 (84.2%)	0.521
	Rural/Isolated	78 (8.2%)	871 (91.8%)		92 (9.7%)	857 (90.3%)		158 (16.7%)	790 (83.3%)	
Years certified	Mean (SD)	24.1 (5.4)	24.0 (5.6)	0.565	24.3 (5.6)	24.0 (5.5)	0.085	24.2 (5.6)	24.0 (5.5)	0.098
Changed specialties	Did not change	206 (8.5%)	2207 (91.5%)	0.614	240 (9.9%)	2174 (90.1%)	0.574	381 (15.8%)	2030 (84.2%)	0.939
	Changed	484 (8.9%)	4944 (91.1%)		516 (9.5%)	4912 (90.5%)		851 (15.7%)	4568 (84.3%)	
Practice area	Primary care	222 (9.1%)	2225 (90.9%)	0.595	242 (9.9%)	2207 (90.1%)	0.449	393 (16.1%)	2052 (83.9%)	0.709
	Surgery — subspecialties	167 (8.6%)	1779 (91.4%)		198 (10.2%)	1750 (89.8%)		295 (15.2%)	1647 (84.8%)	
	Emergency medicine	70 (7.7%)	835 (92.3%)		84 (9.3%)	822 (90.7%)		132 (14.6%)	774 (85.4%)	
	Internal medicine — sub	90 (8.7%)	950 (91.3%)		92 (8.9%)	947 (91.1%)		159 (15.3%)	878 (84.7%)	
	Dermatology	50 (9.1%)	497 (90.9%)		47 (8.6%)	498 (91.4%)		88 (16.1%)	458 (83.9%)	
	Surgery — general	22 (8.1%)	249 (91.9%)		30 (11.1%)	241 (88.9%)		36 (13.3%)	234 (86.7%)	
	Hospital medicine	23 (12.1%)	167 (87.9%)		25 (13.2%)	164 (86.8%)		34 (17.9%)	156 (82.1%)	
	Psychiatry	21 (12.4%)	149 (87.6%)		19 (11.2%)	151 (88.8%)		27 (15.9%)	143 (84.1%)	
	Critical care medicine	8 (7.3%)	101 (92.7%)		6 (5.6%)	102 (94.4%)		14 (12.8%)	95 (87.2%)	
	Other	213 (8.8%)	2202 (91.2%)		222 (9.2%)	2197 (90.8%)		405 (16.8%)	2006 (83.2%)	

As demonstrated in Table 4, ANOVA revealed a significant association between age and whether PAs reported applying learning from PANRE-LA to their clinical practice (*p* = 0.020). Follow-up Scheffe’s post hoc tests showed that the only statistically significant difference (*p* = 0.038) by age was that PAs who indicated applying learning from PANRE-LA to their practice were slightly younger (*M* = 52.9; *SD* = 7.3) vs. those who said no (*M* = 53.4; *SD* = 7.4). Similarly, there was a significant ANOVA result for years of certification with applying learning to practice (*p* = 0.002). Follow-up Scheffe’s post hoc tests determined that PAs who indicated yes were certified for fewer years (*M* = 23.7; *SD* = 5.4) than those who anticipate doing so in the future (*M* = 24.0; *SD* = 5.6; *p* = 0.036) and those who said no (*M* = 24.2; *SD* = 5.6; *p* = 0.005). We also observed that a slightly but statistically significantly higher proportion of PAs who speak another language besides English with their patients than those who communicate only in English responded in the affirmative (41.1% vs. 37.2%; *p* < 0.001). We observed small yet statistically significant associations when analyzing variations across US regions (*p* = 0.022). PAs in the South were most likely to respond affirmatively (39.2%), while those in the Midwest were least likely (34.9%).

**Table 4:** PA demographic and practice characteristics by whether PAs applied learning from PANRE-LA to clinical practice

Characteristic		No (*n* = 2389)	No, but I anticipate doing so in the future (*n* = 4572)	Yes (*n* = 4273)	*p*-value
Age	Mean (SD)	53.4 (7.4)	53.3 (7.4)	52.9 (7.3)	0.020
Gender	Female	1577 (20.9%)	3110 (41.2%)	2863 (37.9%)	0.226
	Male	812 (22.1%)	1461 (39.8%)	1401 (38.1%)	
Race	White	1960 (21.3%)	3746 (40.8%)	3484 (37.9%)	0.957
	Asian	103 (21.6%)	191 (40.0%)	183 (38.4%)	
	Black/African American	90 (22.4%)	155 (38.6%)	157 (39.1%)	
	Other	111 (19.9%)	231 (41.5%)	215 (38.6%)	
Ethnicity	Non-Hispanic/Latino(a)	2154 (21.5%)	4064 (40.6%)	3804 (38.0%)	0.244
	Hispanic/Latino(a)	117 (19.0%)	267 (43.3%)	232 (37.7%)	
Speaks second language	No	1863 (22.0%)	3456 (40.8%)	3150 (37.2%)	< 0.001
	Yes	448 (18.6%)	972 (40.3%)	989 (41.1%)	
US region	South	836 (21.4%)	1535 (39.3%)	1531 (39.2%)	0.022
	Northeast	562 (20.2%)	1149 (41.4%)	1067 (38.4%)	
	West	485 (20.9%)	942 (40.5%)	898 (38.6%)	
	Midwest	500 (22.7%)	933 (42.4%)	769 (34.9%)	
Rural–urban setting	Urban	2188 (21.4%)	4145 (40.5%)	3897 (38.1%)	0.544
	Rural/isolated	191 (20.2%)	399 (42.2%)	356 (37.6%)	
Years certified	Mean (SD)	24.2 (5.6)	24.0 (5.6)	23.7 (5.4)	0.002
Changed specialties	Did not change	517 (21.2%)	1009 (41.5%)	908 (37.3%)	0.614
	Changed	1179 (21.5%)	2206 (40.3%)	2091 (38.2%)	
Specialty	Primary care	508 (20.6%)	964 (39.1%)	994 (40.3%)	0.122
	Surgery — subspecialties	421 (21.5%)	809 (41.4%)	725 (37.1%)	
	Emergency medicine	209 (23.1%)	353 (39.0%)	344 (38.0%)	
	Internal medicine — sub	215 (20.6%)	448 (43.0%)	379 (36.4%)	
	Dermatology	122 (21.8%)	231 (41.2%)	207 (37.0%)	
	Surgery — general	47 (17.2%)	116 (42.3%)	111 (40.5%)	
	Hospital medicine	52 (27.4%)	66 (34.7%)	72 (37.9%)	
	Psychiatry	44 (25.4%)	68 (39.3%)	61 (35.3%)	
	Critical care medicine	19 (16.8%)	56 (49.6%)	38 (33.6%)	
	Other	498 (20.4%)	1002 (41.1%)	938 (38.5%)	

In our last analysis, we evaluated whether demographic and practice characteristics were independent predictors of PAs anticipating or applying their learning to clinical practice (Table 5). Interestingly, the direct multivariate logistic regression showed that age, years certified, and using a second language with patients were not significant predictors. However, male vs. female PAs (adjusted odds ratio (aOR) 0.85, *p* = 0.019), those residing in the Midwest vs. South (aOR 0.84; *p* = 0.032), and PAs practicing in emergency medicine vs. primary care (aOR 0.76; *p* = 0.018) all had lower odds of indicating that they anticipate applying/applied learning to their clinical practice.

**Table 5:** Multivariate Logistic Regression Results: Associations of PA Demographic and Practice Characteristics with Anticipating/Applying Learning From PANRE-LA to Clinical Practice

Variable		95% CI		*p*-value
	OR	LL	UL	
Age	1.01	1.00	1.02	0.252
Gender				
Female (reference)				
Male	0.85	0.75	0.97	0.019
Race				
White (reference)				
Asian	1.07	0.79	1.49	0.669
Black/African American	0.96	0.70	1.35	0.825
Other	0.90	0.67	1.21	0.470
Ethnicity				
Non-Hispanic/Latino(a) (reference)				
Hispanic/Latino	1.03	0.77	1.41	0.836
Speaks second language				
No (reference)				
Yes	1.17	1.00	1.38	0.055
US region				
South (reference)				
Northeast	1.02	0.86	1.19	0.855
West	0.99	0.83	1.17	0.877
Midwest	0.84	0.71	0.99	0.032
Rural–urban setting				
Urban (reference)				
Rural/isolated	1.11	0.91	1.37	0.312
Years certified	0.99	0.98	1.01	0.240
Changed specialties				
Did not change (reference)				
Changed one or more times	0.98	0.86	1.12	0.790
Practice area				
Primary care (reference)				
Surgery—subspecialties	1.00	0.83	1.20	0.978
Emergency medicine	0.76	0.60	0.95	0.018
Internal medicine—subspecialties	1.08	0.86	1.35	0.528
Dermatology	0.86	0.66	1.14	0.285
Surgery—general	1.27	0.86	1.94	0.247
Hospital medicine	0.69	0.46	1.05	0.074
Psychiatry	0.72	0.47	1.12	0.135
Critical care medicine	1.25	0.70	2.41	0.480
Other	1.04	0.87	1.23	0.671

## Discussion

The overall objective of our study was to assess the perspectives and experiences of PAs who have completed the first two quarters of PANRE-LA and determine whether views varied based on a wide variety of demographics and practice characteristics. With a large sample and the power to detect even slight differences, our study found no significant differences in broad perspectives regarding PANRE-LA fostering learning. Over 90% agreed that this assessment provides a learning experience, helps identify gaps in core medical knowledge, provides an opportunity to improve in areas where PAs may have gaps in core medical knowledge, aligns with a lifelong learning perspective, and keeps core medical knowledge up-to-date. Slightly fewer (84.2%) believed it helps them to be a better practitioner. When asked whether PAs have been able to apply anything they learned during PANRE-LA to their clinical practice, the responses were more mixed: 21.3% had not, 40.7% anticipated doing so in the future, and 38.0% confirmed they had already done so. Slight but statistically significant associations with PA characteristics were observed for applying learning to clinical practice.

Our findings are consistent with but also extend prior work conducted by Kozikowski et al. [30], who explored PA perspectives regarding certification. In that study, the majority of PAs viewed certification favorably. For instance, 86% indicated that it helps to update medical knowledge, and 81.4% said it provides objective evidence of continued competence. However, that study revealed that perceptions slightly differed by demographics and practice characteristics. Younger PAs, those underrepresented in medicine and practicing in the primary care discipline, had more positive views. This difference may be due to the study examining the global value of certification for PAs as opposed to the perspectives regarding specific certification exam programs (i.e., initial certification vs. traditional re-certification vs. pilot LA). Additionally, qualitative data from the research suggested that PAs had disparate viewpoints regarding initial certification and MOC. Our study extends the previous findings, given the specific focus on PANRE-LA.

Although LAs developed by various certifying boards share common elements that enhance learning—such as being spaced over time, identifying knowledge gaps, providing immediate feedback with rationales, retesting knowledge gaps, and being administered online—there are some notable differences [19]. These differences include the content being tested, the number of questions, the quarters allotted to complete the assessment, and the time given to respond to questions [3, 15, 19, 22, 23, 25]. For example, CRNAs and anesthesiologists have 1 min to respond to questions on their respective LAs, while PAs, pediatricians, and family medicine physicians have 5 min [3, 15, 19, 22, 23, 25]. However, our findings align with research exploring CRNAs’, pediatricians’, family medicine physicians’, and anesthesiologists’ perspectives on LAs. Choudhry et al. [22] conducted a randomized controlled trial with CRNAs comparing LA vs. traditional assessment for continuing certification. Most CRNAs who completed the LA option indicated that this assessment helped them identify knowledge gaps, promoted lifelong learning, and helped keep knowledge current. Notably, views of CRNAs in the LA condition were more favorable than those in the traditional assessment. Similarly, Turner et al. [23] evaluated pediatrician self-reported learning and practice modification from the Maintenance of Certification Assessment for Pediatrics (MOCA-Peds). The authors found that the majority agreed that MOCA-Peds has helped to identify knowledge gaps. Most anesthesiologists participating in the Maintenance of Certification in Anesthesiology (MOCA-Minute) had positive opinions regarding the program [4]. Similarly, evaluation findings from the Family Medicine Certification Longitudinal Assessment (FMCLA) pilot data with over 11,000 family physicians showed strong support of self-reported learning [24, 25]. A large majority said FMCLA helps with keeping up with medical knowledge. 

Certifying boards gather demographic data and practice information to conduct DIF studies and determine if there are differences by examinee characteristics in perceptions, such as self-reported learning benefits from LAs [26–28]. These types of analyses are important to ensure equity in assessment and that certain groups are not unintentionally disadvantaged. If perceptions of LAs differ significantly by examinee characteristics, it may indicate barriers to engagement and the need to refine assessments to be more equitable. However, prior studies have not extensively explored potential differences in perspectives regarding LAs by provider characteristics. Turner et al. [23] explored pediatrician views of MOCA-Peds by characteristics such as age and certification type, finding that perspectives were more favorable among younger vs. older pediatricians and those in general pediatrics compared to pediatrics subspecialties. However, the study did not explore other personal and professional factors, such as race, ethnicity, and years in practice. Similarly, other work has not explored these potential differences in CRNAs, anesthesiologists, and family medicine physicians [4, 22, 24, 25]. Our work adds to the literature by demonstrating that broad views regarding PANRE-LA fostering learning are uniformly positive among PAs regardless of background, suggesting equitable self-reported learning benefits. However, existing research suggests that provider characteristics might influence specific aspects of LA interaction as well as relevance and confidence ratings [16, 31]. These findings highlight the need for further research to understand provider characteristics associated with LA interactions.

Another finding in our study was that despite favorable overall findings, slightly fewer PAs believed LA helps them become better practitioners. This finding aligns with observations with CRNAs [22], where mean ratings for LA’s impact on patient care and practice modification were lower than other aspects. Similarly, Turner et al. [23] reported that 60.1% of pediatricians agreed that MOCA-Peds helps to provide better patient care, compared to 86.6% who endorsed its role as a helpful learning tool and 77.1% who said it helped to identify knowledge gaps. Likewise, a slightly lower proportion of MOCA Minute diplomates agreed (81%) that the program helps to provide better patient care compared to acknowledging the assessment’s value in staying current in anesthesiology (91%) [4]. For PAs, this disconnect might stem from the PANRE-LA’s emphasis on core medical knowledge, which potentially may be less relevant for the estimated 77% of PAs in non-primary care specialties [32]. However, our analysis did not reveal statistically significant differences by specialty. Further research is necessary to explore the underlying reasons behind this observed difference in positive perceptions of LA fostering learning and slightly lower perceived impact on practice.

Lastly, the finding that only 38% of PAs had applied learning to their clinical practice is most likely due to PANRE-LA participants having only completed their first two quarters. Almost 41% indicated anticipating applying learning from PANRE-LA in the future. A concerning finding in the bivariate analyses was that older PAs and those with more years of experience were slightly but significantly less likely to report that they applied learning to clinical practice. However, these variables were no longer significant after adjustment in the multivariate logistic regression. Conversely, gender and specialties were not statistically significant in bivariate analyses. However, they became so in our multivariable logistic model, likely due to some degree of confounding with other variables only adjusted in the multivariate analysis. Male PAs had 15% lower odds compared to female PAs of indicating either anticipating or already having applied learning from PANRE-LA to their clinical practice. Similarly, PAs practicing in emergency medicine vs. primary care had 24% lower odds. Further research is warranted to explore the underlying reasons for these differences.

Our study acknowledges several limitations that should be considered when interpreting these findings. First, we relied on participants’ self-reported learning rather than measuring objective changes in clinical knowledge resulting from PANRE-LA participation. Second, as with other self-report studies, the results may be influenced by social desirability and recall biases. Third, the modest response rate of 27.7% raises potential concerns regarding sample representativeness. Although the demographic and practice characteristics of survey participants mostly mirrored that of all PAs enrolled in PANRE-LA, survey participants had a higher mean age than non-participants. Furthermore, it is possible that PANRE-LA participants who opted into our survey held more favorable views toward the assessment than those who did not participate. Additionally, our data collection phase was only about 3 weeks. However, during the final week of data collection, we received very few responses, indicating that extending the survey data collection period would likely not have increased the response rate. We did not conduct a power analysis because all PANRE-LA participants were given the opportunity to participate. Assuming a small effect size (*d* = 0.2) and alpha = 0.05, with a large sample size of *n* = 11,896, the power is greater than 99%.

Another limitation is that we dichotomized the scales to facilitate interpretation and enable specific analyses. Although this practice is common, research indicates it can lead to a loss of information and should be approached cautiously [33, 34]. However, dichotomization is justifiable when infrequent observations exist in certain scale categories [35–37]. In our case, because fewer respondents selected “strongly disagree,” “somewhat disagree,” and “neutral,” we combined these categories into “not agree” and merged “somewhat agree” and “strongly agree” into “agree” to enhance interpretability. Additionally, we dichotomized the “Have you been able to apply anything you learned during PANRE-LA to your clinical practice?” question to be able to conduct a multivariate logistic regression, which requires a binary outcome variable. Finally, by assessing participants after they completed the initial two quarters, our study may only reflect preliminary perspectives; their viewpoints may evolve after completing subsequent quarters of the program.

## Conclusion

Evaluating potential differences in examinee characteristics based on self-reported learning from LAs enables certifying boards to identify disparities. This process is essential for refining assessments, promoting equity, and fostering trust among certified professionals and the public. Most PAs across a broad range of demographics and practice characteristics indicated that PANRE-LA helps identify knowledge gaps, provides an opportunity to improve, keeps core medical knowledge up-to-date, and helps to improve practitioner performance. These findings suggest equity in self-reported learning benefits derived from PANRE-LA participation. Further evaluation is warranted to determine if the proportion who applied learning from PANRE-LA to clinical practice increases demonstrably with accumulated participation quarters. LAs provide unique opportunities to promote equity in knowledge acquisition, foster continuous learning, and ultimately contribute to improved patient care.

## Data Availability

The datasets generated and analyzed during the current study are not publicly available due to the confidentiality of individualized data, but de-identified data can be available if requested from the corresponding author.
